# MVCT versus kV‐CBCT for targets subject to respiratory motion: A phantom study

**DOI:** 10.1002/acm2.13356

**Published:** 2021-07-16

**Authors:** Geoffrey Baran, Michael M. Dominello, Todd Bossenberger, Peter Paximadis, Jay W. Burmeister

**Affiliations:** ^1^ Department of Radiation Oncology Karmanos Cancer Institute Detroit MI USA; ^2^ Department of Radiation Oncology Karmanos Cancer Institute and Wayne State University Detroit MI USA; ^3^ Department of Radiation Oncology Lakeland Medical Center Saint Joseph MI USA

**Keywords:** CBCT, image guidance, MVCT, respiratory motion, stereotactic body radiation therapy

## Abstract

The use of kilovoltage cone‐beam computed tomography (kV‐CBCT) or megavoltage computed tomography (MVCT) for image guidance prior to lung stereotactic body radiation therapy (SBRT) is common clinical practice. We demonstrate that under equivalent respiratory conditions, image guidance using both kV‐CBCT and MVCT may result in the inadequate estimation of the range of target motion under free‐breathing (FB) conditions when standard low‐density window and levels are used. Two spherical targets within a respiratory motion phantom were imaged using both long‐exhale (LE) and sinusoidal respiratory traces. MVCT and kV‐CBCT images were acquired and evaluated for peak‐to‐peak amplitudes of 10 or 20 mm in the cranial‐caudal direction, and with 2, 4 or 5 s periods. All images were visually inspected for artifacts and conformity to the ITV for each amplitude, period, trace‐type, and target size. All LE respiratory traces required a lower threshold HU window for MVCT and kV‐CBCT compared to sinusoidal traces to obtain 100% volume conformity compared with the theoretical ITV (ITV_T_). Excess volume was less than 2% for all kV‐CBCT contours regardless of trace‐type, breathing period, or amplitude, while the maximum excess volume for MVCT was 48%. Adjusting window and level to maximize conformity with the ITV_T_ is necessary to reduce registration uncertainty to less than 5 mm. To fully capture target motion with either MVCT or kV‐CBCT, substantial changes in HU levels up to −600 HU are required which may not be feasible clinically depending on the target's location and surrounding tissue contrast. This registration method, utilizing a substantially decreased window and level compared to standard low‐density settings, was retrospectively compared to the automated registration algorithm for five lung SBRT patients exposed to pre‐treatment kV‐CBCT image guidance. Differences in registrations in the super‐inferior (SI) direction greater than the commonly used ITV to PTV margin of 5 mm were encountered for several cases. In conclusion, pre‐treatment image guidance for lung SBRT targets using MVCT or kV‐CBCT is unlikely to capture the full extent of target motion as defined by the ITV_T_ and additional caution is warranted to avoid registration errors for small targets and patients with LE respiratory traces.

## 1 INTRODUCTION

Medically inoperable stage I/II non‐small cell lung cancers (NSCLC) are often treated with stereotactic body radiation therapy (SBRT) providing local control rates comparable to surgery.^1^, ^2^, ^3^, ^4^, ^5^, ^6^, ^7^, ^8^ Due to the higher risks associated with large doses per fraction, steep dose gradients, and reduced margins with SBRT, geometric target accuracy is critical. The use of on‐board imaging (OBI) kilovoltage cone‐beam computed tomography (kV‐CBCT) or megavoltage computed tomography (MVCT) prior to delivery has dramatically improved the spatial accuracy of treatments and allowed for planning target volume (PTV) margin reduction.^9^, ^10^, ^11^, ^12^, ^13^, ^14^ The question remains whether kV‐CBCT or MVCT accurately characterize the complete range of target motion under free‐breathing (FB) conditions. If patient breathing characteristics at the time of treatment are assumed to be the same as during 4DCT acquisition, one might expect the planned internal target volume (ITV) to match the pre‐treatment target volume as measured with OBI. However, underrepresentation of this target excursion, due to the poor selection of window and level or respiratory trace characteristics (e.g., long‐exhale (LE)), combined with differences in image acquisition speeds, has the potential to lead to incorrect positioning for treatment.^15^, ^16^, ^17^, ^18^, ^19^, ^20^, ^21^

Megavoltage computed tomography, as utilized by tomotherapy (Accuray, Sunnyvale, CA), uses 3.5 MV x‐rays for image guidance, resulting in reduced contrast and greater extraneous noise compared to kV‐CBCT.^22^, ^23^ Given the slow MVCT acquisition time of 5 s per slice, moving targets appear blurred. The appearance of a mobile target captured with MVCT was investigated by Smeenk et al, who used a sinusoidal trace with peak‐to‐peak amplitude of 10 mm and a breathing period of either 4.0 or 1.0 s was used to drive a target in the lateral or cranial‐caudal direction. The results established that MVCT under typical breathing conditions does not fully capture target motion. Motion artifacts were worse when the motion trajectory was in the lateral direction albeit at an amplitude and direction unlikely to be observed clinically. For a clinically unrealistic breathing period of 1.0 s to simulate the benefit of an ultra‐slow scan, motion artifacts were reduced and the image better represented the ITV known a priori in both volume and shape.^18^ Goosens et al. studied the use of tumor‐based registration between average planning kVCT and MVCT under clinically relevant respiration conditions in all three anatomical directions and found a high degree of registration accuracy except when the tumor motion period was equal to half of the gantry period during MVCT acquisition due to aliasing artifacts.^21^ These studies made use of simple sinusoidal motion, as opposed to the LE motion generally observed clinically.

Kilovoltage cone‐beam computed tomography should more accurately capture a target's full excursion compared to MVCT due to the conical acquisition of projections of the entire target for multiple respiratory cycles and the improved contrast achieved by a lower energy beam. As demonstrated by Vergalasova et al., the use of kV‐CBCT does not entirely eliminate the underrepresentation of an ITV, as underestimates of up to 40.1% were realized as compared to an ITV generated with 4D‐CBCT based on a patient's respiratory trace.^15^ Similar results were seen by Clements et al., with the added remark that if patient breathing characteristics are known, it might be more appropriate to minimize potential shift errors when the ITV is underrepresented by kV‐CBCT by an edge‐to‐edge alignment technique of the target as opposed to center‐to‐center under irregular patient breathing conditions.^16^ This would require monitoring respiration during kV‐CBCT acquisition which is often not feasible in clinical practice and would be prone to debate when registering the volume to a superior or inferior edge instead of the center, depending on inhale to exhale conditions. For 71 lung SBRT patients, Liu et al. found the ITV to be underrepresented by 11.8% (−49.8 to 24.3%) on average from kV‐CBCT compared to 4DCT with central lesions being more likely than peripheral to be smaller than the volume contoured on 4DCT. However, this may be a result of reduced contrast when a lesion is close to the mediastinum and not a result of the inability of kV‐CBCT to capture the complete motion trajectory of a target.^17^ This study also made use of a standard window and level setting. We propose that this needs to be changed dynamically depending on the target's shape, motion trajectory, size, and local anatomy.

To our knowledge, a direct comparison between kV‐CBCT and MVCT under controlled and equivalent breathing conditions has yet to be explored. We hypothesize that kV‐CBCT and MVCT will both underestimate a target's true trajectory at clinically used low‐density window and level settings, but kV‐CBCT has the capability to provide improved soft‐tissue registration compared to MVCT when window and level are optimized for the evaluation of target excursion.

## 2 MATERIALS AND METHODS

**FIGURE 1:**
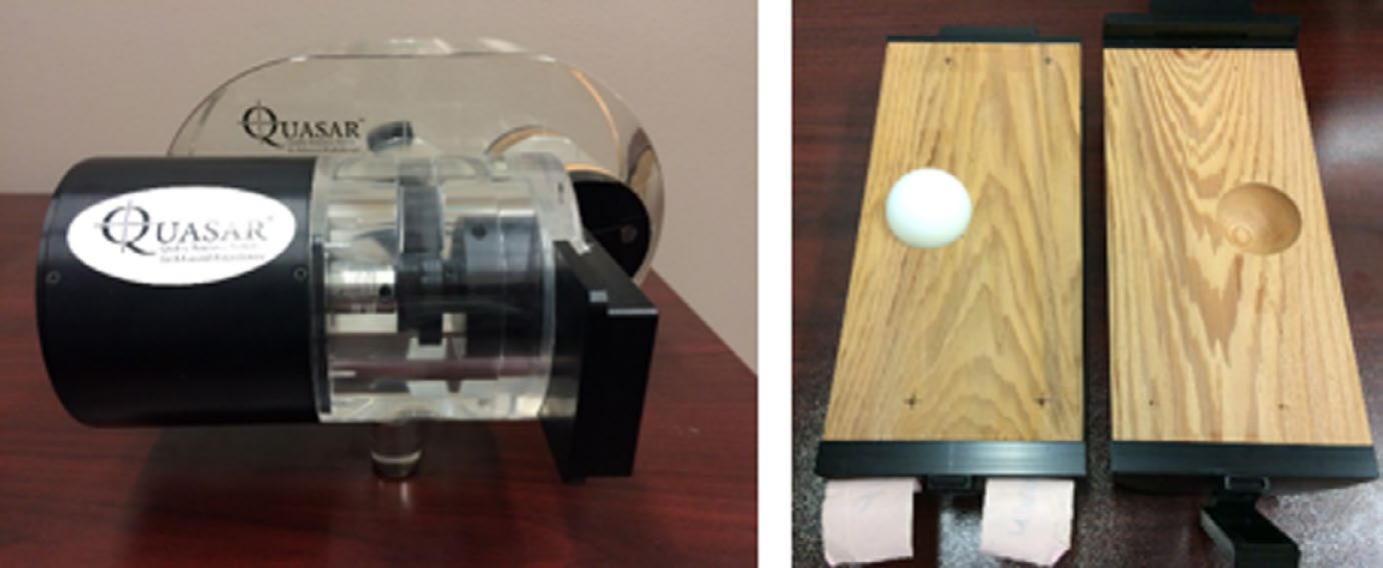
Quasar respiratory motion phantom with 30 mm polystyrene spherical target

The Quasar respiratory motion phantom (Modulus Medical Devices Inc., London, ON), as seen in Figure 1, was used to evaluate MVCT and kV‐CBCT image quality. The phantom consists of a 30 × 20 × 12 cm^3^ acrylic body, in which a movable 8 cm diameter cylindrical lung‐equivalent cedar insert is embedded. The cedar insert, containing either a 30 or 15 mm diameter polystyrene spherical target, is fixed to a drive unit to allow for one‐dimensional motion. The Quasar respiratory motion software (Modulus Medical Devices Inc., London, ON) was used to drive the tumor with two respiratory traces of 10 or 20 mm amplitude in the cranial‐caudal direction and 2, 4, or 5 s periods. A depth gauge was used to confirm the accuracy of the software‐controlled amplitude and maintain the expected value to within ±0.2 mm. Peak‐to‐peak amplitudes and periods were chosen based on common clinical ranges and to be comparable to the motion parameters used by previous investigators.^24^, ^25^, ^26^ The simple sinusoidal motion was first used to facilitate direct comparisons to previous work, while a second trace with an extended exhale period compared to inhale was used to determine whether this more clinically appropriate trace would have an impact on MVCT or kV‐CBCT target registration.

Megavoltage computed tomography images were acquired with Tomotherapy HI‐Art's 3.5 MV x‐ray beam. The phantom was positioned at the planned isocenter by aligning fiducial markers on the phantom's surface with the treatment room's external lasers. A digital level was used to limit any phantom tilt in both the superior–inferior and left–right directions to less than 0.2°. A 2 mm slice width was used for all scans and the scan length was chosen to obtain an MVCT that would encompass the entirety of the expected motion envelope plus a 3–4 slice (6–8 mm) margin from the superior and inferior borders of the theoretical ITV. Two slices per 10.0 s gantry rotation period were acquired. All scans were reconstructed with a slice spacing of 2 mm following the current lung SBRT protocol at our institution. The reconstructed MVCT slices had a field‐of‐view (FOV) of 40 cm.

Kilovoltage cone‐beam computed tomography images were acquired with the OBI on Varian's TrueBeam linear accelerator (Varian Medical System, Palo Alto, CA). Similar to the MVCT set‐up, the phantom was positioned at the linac's isocenter using the external lasers. The thorax imaging protocol was used with beam parameters of 125 kVp and 270 mA s, with 900 projections obtained over the full 360° rotation while the gantry rotated at 6° per second. The reconstructed images had a slice thickness of 1 mm and matrix size of 512 × 512.

**FIGURE 2:**
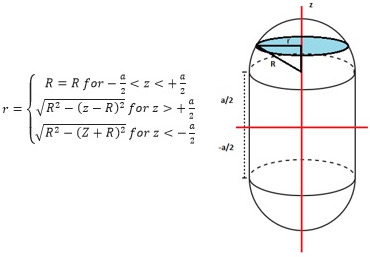
Cross‐sectional radius, r, of the ITV_T_ changes as a function of slice position, z, for a given peak‐to‐peak amplitude a, and target radius, R

All MVCT and kV‐CBCT images were exported to Varian's Eclipse treatment planning system (Varian Medical System, Palo Alto, CA) for further analysis. MVCT and kV‐CBCT images were both displayed at the current low‐density window and level used clinically at our institution with a window of 700 and a level of −600 HU. Since the target is moving only in the SI direction, the change in radius (listed in equation as r) of ITV_T_ as a function of slice position (listed in equation as z) can be theoretically determined based on the equation in Figure 2 given a particular peak‐to‐peak amplitude (listed in equation as a). An ITV_T_ was created as a structure in Eclipse based on shape in Figure 2 and measured volumes were within 1% of theoretical volumes.

Megavoltage computed tomography and kV‐CBCT images were visually inspected for artifacts and underrepresentation. Underrepresentation is defined as the lack of visual or quantitative agreement between what is observed as a target on MVCT or kV‐CBCT compared to ITV_T_. Similar to the assessments performed by Smeenk et al., volume conformity and excess volume^18^ were plotted as a function of threshold HU for each amplitude, period, trace‐type, and target size.

$$Volume\;Conformity=\;\frac{{ITV}_{T}\;\left(cc\right)\cap ITV\;at\;Threshold\;HU\;\left(cc\right)}{{ITV}_{T}\;\left(cc\right)}$$

$$Excess\;Volume=\;\frac{ITV\;at\;Threshold\;HU\;\left(cc\right)‐{\mathrm{ITV}}_{T}\;\left(cc\right)}{{\mathrm{ITV}}_{T}\;\left(cc\right)}$$

Volume conformity, as defined above, is essentially the percentage of the target volume realized by imaging at a given threshold HU (ITV at Threshold HU in equation above) that overlaps with ITV_T_ as defined by Figure 2. If the imaging system perfectly imaged the full range of target motion, volume conformity would have an upper bound of 1. To obtain the ITV at Threshold HU, the target was manually contoured for a given threshold HU by only including the contiguous voxels which had HU values at or greater than a particular threshold HU. Due to image‐induced artifacts, potential misalignments, and lack of contrast, the manually contoured volume may expand beyond the boundaries of the known ITV_T_. The amount of target represented by the on‐board imaging system that escapes the ITV_T_ is measured with the use of excess volume which subtracts voxel‐by‐voxel any contoured regions outside of ITV_T_. Volume conformity and excess volume were plotted as a function of threshold HU for each imaging system. All conformity plots were fit with a linear function to determine the threshold HU necessary to fill ITV_T_ (i.e., 100% conformity).

For a sinusoidal target, as threshold HU increases the target contour should grow symmetrically about the motion envelope origin such that the center of the volume remains at (0, 0, 0). Targets subject to LE motion will have the higher density portion of the visible target located toward the superior border of ITV_T_. Consequently, at a given threshold HU, the target's center of volume coordinate will be greater than 0 along the direction of motion. As threshold HU is increased, this number should approach 0 as the visible target fills the ITV_T_. For a given scan, the shift of the centroid as described above, is recorded as a function of threshold HU.

For the techniques above, threshold HU was reduced in −50 HU increments until the target could no longer be easily discerned from the background. This occurred at a faster rate for MVCT due to reduced contrast, increased noise, and the presence of artifacts. For kV‐CBCT, HU could be decreased to a level that allowed for nearly complete or complete filling of the ITV_T_.

Manual registration using a substantial decrease in window and level compared to automated standard low‐density registrations was retrospectively compared for five lung SBRT patients exposed to pre‐treatment kV‐CBCT image guidance to the proposed registration protocol. Differences in the proposed shifts for the lateral, longitudinal, and vertical directions were measured between the two registration methods. The total target motion for each patient was investigated based on the 4DCT simulation.

## 3 RESULTS

**FIGURE 3:**
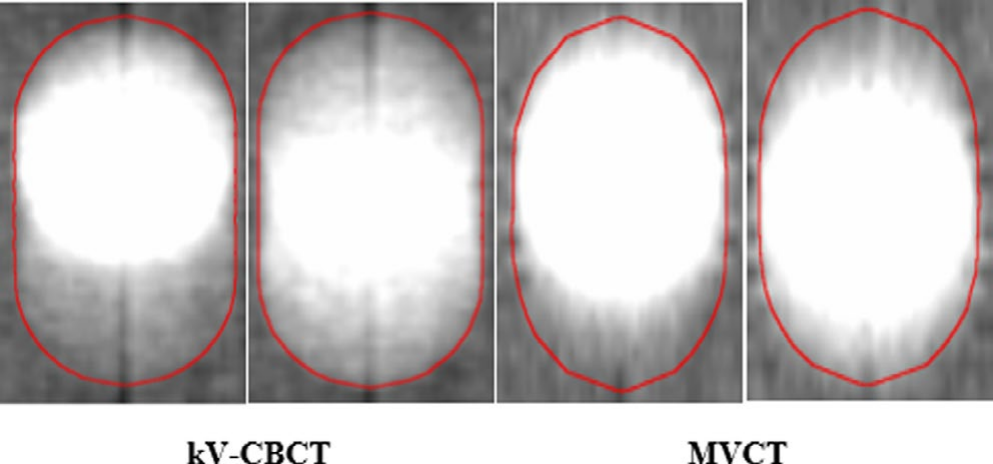
Underrepresentation of target volume (diameter = 30 mm) at a clinically used window and level setting (W = 700 HU, L = −600 HU) for breathing period of 4.0 s and 10 mm peak‐to‐peak amplitude for LE (left) and sinusoidal (right) trace‐types

As observed in Figure 3, the excursion of the spherical target under respiratory motion does not fill the entirety of the ITV outlined in red at the standard low‐density window and level setting. This is true independent of imaging modality, target size, amplitude, period or the characteristics of the respiratory trace. The location of the high‐intensity region depends on the time spent in exhale versus inhale phase. ITV_T_ for MVCT images is not as sharply defined given the use of a 2 mm slice width as compared to the 1 mm slice width used for kV‐CBCT acquisitions.

**FIGURE 4:**
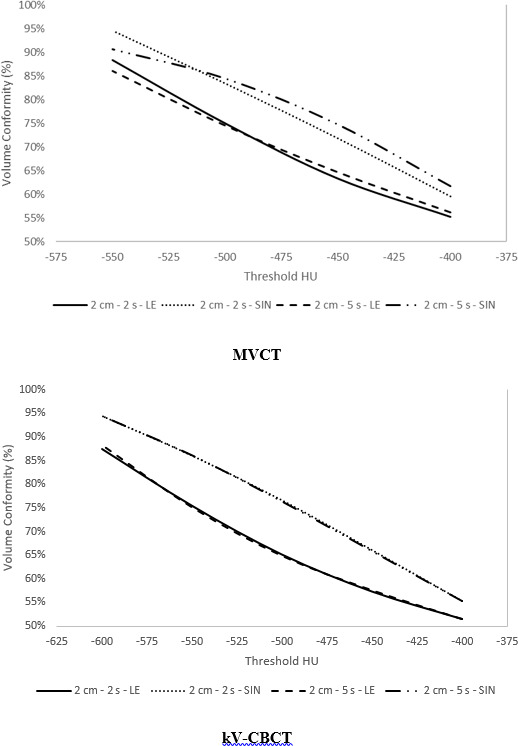
Volume conformity as a function of threshold HU for megavoltage computed tomography and kilovoltage cone‐beam computed tomography for a large target at a peak‐to‐peak amplitude of 20 mm, moving with either sinusoidal or LE motion at a breathing period of 2.0 or 5.0 s

Linear regression analysis found a correlation coefficient greater than 0.95 between volume conformity and threshold HU for MVCT and kV‐CBCT. The threshold HU required to fill ITV_T_ averaged over all breathing periods is summarized in Table 1. For the large target, all MVCT (HU_LE_ – HU_SIN_ = −89 to −21 HU) and kV‐CBCT (HU_LE_ – HU_SIN_ = −73 to −47 HU) scans required a lower threshold HU to fill the ITV for LE scans compared to sinusoidal. Similarly, 5/6 MVCT (HU_20mm_ – HU_10mm_ = −57 to 21 HU) and all kV‐CBCT (HU_20mm_ – HU_10mm_ = −46 to −14 HU) scans required a lower threshold HU to fill the ITV for 20 mm compared to 10 mm amplitude. Volume conformity is plotted as a function of threshold HU (Figure 4) for a peak‐to‐peak amplitude of 20 mm and breathing periods of 2 and 5 s for MVCT and kV‐CBCT, respectively.

**TABLE 1:** HU necessary to fill ITV_T_ as a function of peak‐to‐peak amplitude, respiratory trace type, and imaging modality

Amplitude	Respiratory trace	Fill HU	
		MVCT	kV‐CBCT
1 cm	SINE	−558	−599
	LE	−604	−655
2 cm	SINE	−578	−617
	LE	−626	−685
		

**FIGURE 5:**
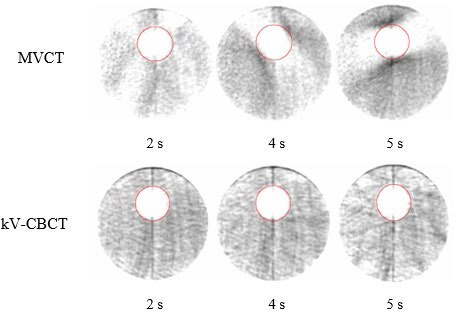
Off‐axis transverse slice of the target showing the difference in aliasing artifacts for megavoltage computed tomography compared to kilovoltage cone‐beam computed tomography

**FIGURE 6:**
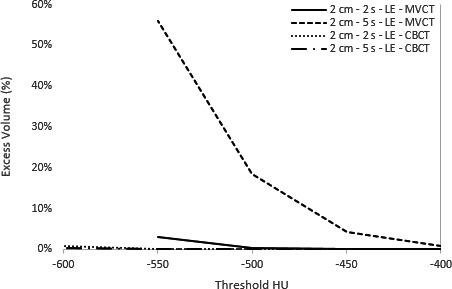
Excess volume as a function of threshold HU for megavoltage computed tomography versus kilovoltage cone‐beam computed tomography for a peak‐to‐peak amplitude of 20 and 30 mm target size

Under equivalent motion conditions, Figure 5 presents a visual comparison of the target in the axial plane for MVCT versus kV‐CBCT. The presence of artifacts is dependent on the breathing period. The volume outside of ITV_T_ for a given HU threshold was measured. Figure 6 shows that the excess volume is plotted as a function of HU for breathing periods of 2.0 and 5.0 s for a peak‐to‐peak amplitude of 20 mm. It is important to note the difference in the excess volume scale when comparing images subject to 2.0 s breathing motion compared to 5.0 s. For the large target, excess volume ranged from 0.2 to 17.0% at −600 HU and 2.3 to 65.7% at −550 HU for kV‐CBCT and MVCT, respectively. Excess volume was <2% for all kV‐CBCT contours regardless of trace type, breathing period or amplitude. In contrast, excess volume was largest for a period of 5.0 s (average of 48%), followed by 4.0 s (average of 25%) but reduced for 2.0 s (average of 5%) for MVCT.

Table 2 summarizes the centroid shift at a threshold HU of −400 and −550 HU for MVCT and −400 and −600 HU for kV‐CBCT. As mentioned, −550 and −600 HU are the minimum levels that can be applied without compromising target visibility compared to the background for MVCT and kV‐CBCT, respectively. If the high‐density region is mistakenly placed in the middle of the ITV_T_ for LE respiration, this would result in a registration error similar to the value presented. At higher threshold values (i.e., −400 HU), the centroid shift is significantly greater for LE scans (6.4 and 4.8 mm for MVCT and kV‐CBCT, respectively) due to the presence of greater HU values at the superior aspect of ITV_T_. All sinusoidal centroid shifts were less than 2 mm (0.1 to 2 mm).

**TABLE 2:** Averaged centroid shift over all breathing periods at a threshold HU of −400 and −550 HU for megavoltage computed tomography (MVCT) and −400 and −600 HU for kilovoltage cone‐beam computed tomography (kV‐CBCT)

Amplitude	Respiratory trace	SI centroid shift (mm)			
		MVCT		kV‐CBCT	
		−400 HU	−550 HU	−400 HU	−600 HU
1 cm	LE	1.9	1.6	2.1	0.6
2 cm	LE	6.4	3.1	4.8	1.2

Scans were replicated for MVCT with a target size of 15 mm with similar results. All MVCT (HU_LE_ – HU_SIN_ = −111 to −40 HU) scans required a lower threshold HU to fill ITV_T_ for LE scans compared to sinusoidal. A smaller average difference in threshold HU was required for the small target compared to large (HU_small_ – HU_large_ = −64 to 30 HU) for 10/12 cases. Excess volume for the smaller target showed a similar dependence on breathing period: 5.0 s (average = 44%), 4.0 s (average = 21%), and 2.0 s (average = 3%). The potential for registration error was increased by approximately 1.0 mm on average compared to the large target (Table 3).

**TABLE 3:** Fill HU and SI centroid shift as a function of the target size for peak‐to‐peak amplitudes of 10 and 20 mm

Target size	Amplitude (mm)	Fill HU	SI centroid shift (mm) −400 HU	SI centroid shift (mm) −550 HU
15 mm	10	−618	3.2	2.5
	20	−669	7.5	5.0
30 mm	10	−604	1.9	1.6
	20	−626	6.4	3.1

The difference between automated registration and the registration technique presented here was evaluated and averaged over five fractions for each of five lung SBRT patients. The results are presented in Table 4 and include the target size and total target motion. Differences were minimal in the vertical and lateral directions with all averages less than 0.25 cm. In contrast, longitudinal differences up to 0.85 cm were observed with an average difference of 0.55 cm for all fractions for one patient.

**TABLE 4:** Average differences in registration values in the lateral, longitudinal, and vertical direction when comparing automated registration to manual registration using drastic changes in window and level to attempt to fill the ITV for five lung SBRT patients

Patient	Target size (cc)	Target motion (cm)	Δ Lat. (cm)	Δ Long. (cm)	Δ Vert. (cm)
1	0.46	0.9	−0.01 ± 0.05	0.55 ± 0.21	0.00 ± 0.08
2	4.83	1.2	−0.02 ± 0.05	−0.31 ± 0.11	0.03 ± 0.05
3	2.41	0.5	0.25 ± 0.07	0.36 ± 0.02	−0.05 ± 0.09
4	2.46	1.2	0.00 ± 0.18	−0.16 ± 0.10	0.04 ± 0.04
5	1.70	0.9	−0.21 ± 0.08	0.40 ± 0.14	0.00 ± 0.01

## 4 DISCUSSION

**FIGURE 7:**
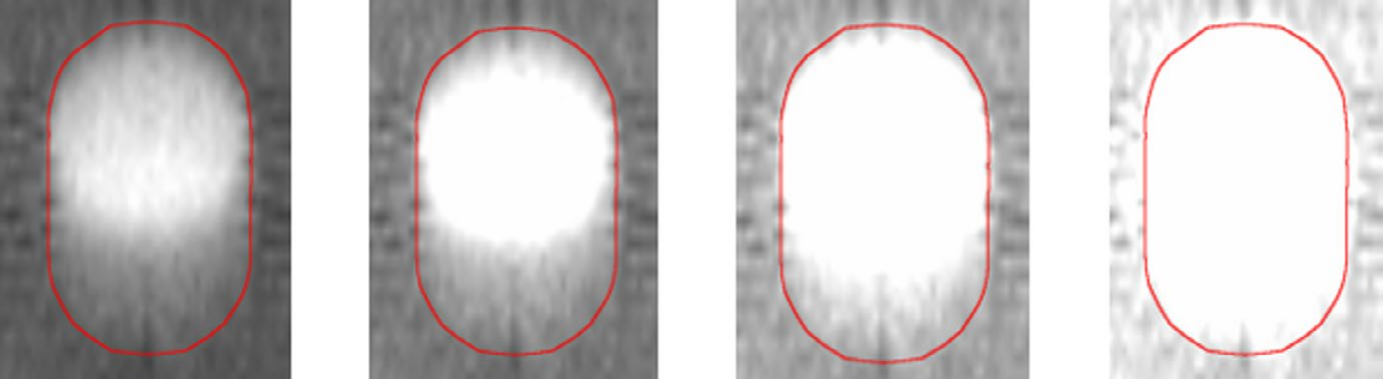
Coronal slice of target motion showing that as window and level is decreased, target appearance grows inferiorly for long‐exhale (LE) scans

As predicted, neither imaging modality captures the complete target excursion at standard low‐density window and levels (Figure 3). In agreement with the literature, this is especially true when the target spends minimal time at a given position, for example, when a relatively short amount of time is spent at full inhalation.^15^ As demonstrated by Clements et al, a potential incorrect target shift could be made when using a center‐to‐center registration technique with the planning ITV.^16^ This can be minimized by placing the underrepresented target appearance at a particular edge of the ITV if breathing characteristics are known a priori. As this knowledge is often unlikely to be available clinically, an alternative is to attempt to fill ITV_T_ as window and level are decreased as shown in Figure 7. The direction of target growth on MVCT or kV‐CBCT as level is decreased assists in understanding the patient's breathing pattern during image acquisition. If there is equivalent bi‐directional growth above and below the target as the level is decreased, the patient is breathing with a more sinusoidal respiratory trace. In contrast, growth in a single direction is indicative of breathing with long inhalation or exhalation periods.

Based on the phantom results in Table 1, decreasing HU level to −600 HU or less is required to approach 100% volume conformity. Unfortunately, even under ideal phantom conditions, the target cannot be distinguished accurately using MVCT due to the lack of contrast between the target and cedar lung insert. This is not true for kV‐CBCT given the lower acquisition energy.

Compared to a phantom, lung tumors come in various sizes, shapes, locations, and densities. Centroid shifts or registration errors were observed up to 6.4 and 4.8 mm at a threshold HU of −400 HU for MVCT and kV‐CBCT, respectively, for LE motion approaching 2 cm. These are reduced to 3.1 and 1.2 mm if threshold HU is further decreased to −550 and −600 HU, respectively. Such decreases are often not clinically feasible since real targets are often smaller, less uniformly shaped, less clearly defined, and likely to be surrounded by denser structures such as chest wall or mediastinum. Consequently, the aforementioned approach of filling ITV_T_ to avoid shift errors is limited in clinical scenarios. However, judicious optimization of window and level to maximize the conformity of ITV_T_ should minimize errors, as observed in Table 2, and keep them within typical inter‐observer registration errors.^27^

As observed in Table 4, manual registration using the technique described here results in substantial differences in the longitudinal direction compared to auto‐registration. Patients with motion approaching 1 to 2 cm were chosen for evaluation as these patients are most likely to benefit from the improved registration technique described here. Since a primary goal in lung SBRT planning is to provide rapid dose fall‐off away from the PTV, even small registration errors where the target marginally falls outside of the PTV can be detrimental, even for a single fraction, and may result in a potential reduction of local control. Averaging the data from Table 4 over all patients yields an average longitudinal difference magnitude of 3.6 mm. While this is within a typical ITV to PTV margin, it effectively reduces the safety margin that accounts for intra‐fraction motion to less than 2 mm if starting with a pre‐existing systematic error.

Any small improvements in target localization are valuable when attempting to reduce the ITV to PTV margin. Depending on the target size, reducing the margin from 5 to 4 mm reduces the volume of normal lung irradiated to high doses and decreases the potential overlap of the PTV with surrounding OARs (esophagus, spinal cord, great vessels, brachial plexus, heart, etc.) that are subjected to maximum dose constraints for lung SBRT prescription regimens. This is increasingly important as a decrease in overlap with OARs may assist in avoiding highly modulated plans to meet constraints that are subjected to increased interplay effects with target motion and longer treatment times. Establishing reduced safety margins without compromising tumor control may be achieved by understanding tumor motion prior to treatment using 4D kV‐CBCT, adapting per‐patient margins depending on breathing characteristics or ability to coach consistent respiration, improving image quality, or decreasing inter‐observer and intra‐observer uncertainty by a standardized registration approach, such as the one presented here.

Consistent with the literature, MVCT images of targets subject to respiratory motion with breathing periods approaching or equal to 5.0 s suffer from aliasing artifacts but as the breathing period decreases, these artifacts are reduced.^21^ This is clearly displayed in Figure 6, where the 5.0 s MVCT image has an excess volume of more than 50% but for 2.0 s is reduced to approximately 5%. These values are not as severe as the results presented by Smeenk et al, albeit with a phantom construction that made use of Styrofoam © as opposed to cedar allowing for increased contrast and further reduction in threshold HU. Compared to previous studies, only SI motion was considered, the direction known to have the largest excursion for lung tumors. Lateral motion is known to increase the severity of aliasing artifacts for MVCT but this increase occurs significantly only at amplitudes unlikely to occur clinically. As seen in Figure 5, artifacts typically extend in the transverse plane off‐axis positions which may not only lead to increased uncertainty in registration errors along non‐SI directions, but also compromise the visualization of surrounding organs‐at‐risk (OARs). No additional high‐density region is seen extending out of ITV_T_ in the direction of motion. No significant motion artifacts were seen for kV‐CBCT outside of a clearly distinguishable windmill effect that would not have an impact on localization as it is easily identifiable compared to the target.

Small targets with large peak‐to‐peak motion may be better visualized with Tomotherapy using an adapted MVCT protocol with longer gantry period or reduced pitch as suggested by Smeenk et al., or a 4D MVCT cine approach, oversampling projection data at each couch position for improved acquisition of the target at all respiratory phases as suggested by Mahan et al.^28^ Such modified techniques may improve the visualization of the target within the entirety of the ITV, decrease artifacts, and consequently decrease potential shift errors to those predicted by kV‐CBCT. 4D kV‐CBCT has become an available pre‐treatment imaging technique to obtain a respiratory‐correlated image of the tumor prior to or, more recently, during delivery.^29^, ^30^, ^31^ Spatial and temporal information about the target trajectory allows making adaptive changes when taking into account average target position and total target amplitude compared to that observed on 4DCT during simulation. For kV‐CBCT, incorrect target shifts (<5 mm here) are unlikely to compromise target coverage even when using standard lung window and levels if target trajectory is assumed constant. Multiple studies have shown that tumor motion at the time of treatment with the use of 4D kV‐CBCT may not match the tumor motion from planning, with increased target trajectories on the order of even 10 mm in a single direction.^32^ As this may not be realized with traditional kV‐CBCT, coverage would be compromised even further when combining this increase in total target motion with an incorrect alignment of the target within the ITV, emphasizing the need for decreased window and level to more fully visualize the entire ITV.

Future work is still required to minimize the uncertainties involved in the safe delivery of SBRT. These include but are not limited to 4D dose predictions, target motion management, patient respiratory consistency, improvements in image quality, real‐time OBI during delivery, automated registration, and adaptive planning. Since MVCT and kV‐CBCT are common pre‐treatment imaging modalities used prior to SBRT delivery, a direct comparison under equivalent target motion conditions was necessary. For both modalities, as threshold HU decreased, the volume conformity of ITV_T_ approached 100%, but these HU values are unlikely to be appreciated clinically due to the lack of contrast, especially for MVCT. In addition, MVCT scans with breathing periods equal to or approaching 5.0 s suffer from aliasing artifacts which may further challenge image registration accuracy. Under the same motion conditions, kV‐CBCT is not affected by such artifacts. By choosing an appropriate window and level or observing the appearance of the target on MVCT or kV‐CBCT as level is changed, potential registration errors can be minimized for LE respiration to values that are less than or approach typical ITV to PTV margins. Both MVCT and kV‐CBCT are adequate for lung SBRT registration but caution should be taken if target trajectory is known to follow LE motion with large amplitudes, if reduced margins are being considered for treatment, or if patient breathing characteristics are known to be inconsistent between simulation and any treatment fraction.

## 5 CONCLUSION

In conclusion, neither MVCT nor kV‐CBCT image guidance techniques are able to capture the full extent of target motion when standard low‐density window and level values are used. MVCT and kV‐CBCT images were acquired and evaluated for simulated tumor motion with various amplitudes and periods. These images were visually inspected for artifacts and conformity to the ITV for each amplitude, period, trace‐type, and target size. The presence of artifacts outside the contoured ITV was observed to be highly dependent on the breathing period. Imaging level was reduced in an attempt to observe the visible target completely fill the known ITV and HU values of −550 and −600 HU were the minimum levels that could be applied without compromising target visibility compared to the background for MVCT and kV‐CBCT, respectively. At level values higher than these thresholds, a target centroid shift is observed, particularly for long‐exhale respiration. In such cases, centroid shifts of 6.4 and 4.8 mm were observed for MVCT and kV‐CBCT, respectively. The difference between automated registration and the registration technique presented here was evaluated and longitudinal differences up to 0.85 cm were observed. These results are relatively large in comparison to typical ITV to PTV margin sizes. While both MVCT and kV‐CBCT can provide accurate pre‐treatment image guidance for lung SBRT registration, judicious optimization of window and level values to maximize the visualized target excursion should minimize registration errors, and keep them within typical ITV to PTV margins. Furthermore, caution should be taken if target trajectory is known to have long‐exhale characteristics with large amplitude or if patient breathing characteristics are known to be inconsistent between simulation and any treatment fraction.

## CONFLICT OF INTEREST

No conflicts of interest.

## AUTHOR CONTRIBUTION

Geoffrey Baran, MS – Corresponding author, Contributed to all methods used for acquiring measurements, collection of all results, analysis of results, and interpretation of the data. Drafted the manuscript. Approved the final work to be published. Made all appropriate edits when necessary. Michael Dominello, DO, Contributed to the design and concept of the project and clinical interpretation of the data. Assisted in writing portions of the manuscript and making major edits. Approved the final work to be published. Todd Bossenberger, MS, Contributed to the design and concept of the project including making preliminary measurements. Made suggestions regarding the results. Added to and reviewed the manuscript. Approved the final work to be published. Peter Paximadis, MD, Contributed to the design and concept of the project include all very preliminary project goals and results. Provided clinical expertise regarding the results and personal opinion on image guidance for each modality used. Reviewed manuscript and approved the final work to be published. Jay W. Burmeister, PhD, Contributed to the design and concept of the projection, collection of results, and interpretation of the results. Assisted in writing portions of the manuscript and making major edits. Approved the final work to be published.
